# Alginate Modified Magnetic Polypyrrole Nanocomposite for the Adsorptive Removal of Heavy Metal

**DOI:** 10.3390/polym15214285

**Published:** 2023-10-31

**Authors:** Fouzia Mashkoor, Mohd Shoeb, Changyoon Jeong

**Affiliations:** School of Mechanical Engineering, Yeungnam University, Gyeongsan 38541, Republic of Korea; fmashkoor@yu.ac.kr (F.M.); mshoeb@yu.ac.kr (M.S.)

**Keywords:** polypyrrole, adsorption, mercury, water pollution, nanocomposite

## Abstract

The presence of heavy metals with high acute toxicity in wastewater poses a substantial risk to both the environment and human health. To address this issue, we developed a nanocomposite of alginate-encapsulated polypyrrole (PPy) decorated with α-Fe_2_O_3_ nanoparticles (Alg@Mag/PPy NCs), fabricated for the removal of mercury(II) from synthetic wastewater. In the adsorption experiments, various parameters were examined to identify the ideal conditions. These parameters included temperature (ranging from 298 to 323 K), initial pH levels (ranging from two to nine), interaction time, amount of adsorbent (from 8 to 80 mg/40 mL), and initial concentrations (from 10 to 200 mg/L). The results of these studies demonstrated that the removal efficiency of mercury(II) was obtained to be 95.58% at the optimum pH of 7 and a temperature of 303 K. The analysis of adsorption kinetics demonstrated that the removal of mercury(II) adhered closely to the pseudo-second-order model. Additionally, it displayed a three-stage intraparticle diffusion model throughout the entire adsorption process. The Langmuir model most accurately represented equilibrium data. The Alg@Mag/PPy NCs exhibited an estimated maximum adsorption capacity of 213.72 mg/g at 303 K, surpassing the capacities of most of the other polymer-based adsorbents previously reported. The thermodynamic analysis indicates that the removal of mercury(II) from the Alg@Mag/PPy NCs was endothermic and spontaneous in nature. In summary, this study suggests that Alg@Mag/PPy NCs could serve as a promising choice for confiscating toxic heavy metal ions from wastewater through adsorption.

## 1. Introduction

With an unprecedented industrial growth rate, the stress on aquatic ecosystems is increasing [1]. Effluents from various industries, including leather processing, textile manufacture, electroplating, metal finishing, and dye production, include dyes, heavy metals, insecticides, pesticides, and other contaminants that contribute significantly to environmental contamination [2,3,4,5]. Heavy metal ions, such as mercury(II), are especially worrying among these pollutants because they are dumped into aquatic ecosystems, presenting significant threats to human health and the ecosystem [6]. Mercury(II) compounds are known to be carcinogenic and can injure critical human organs, resulting in neurological damage, cardiovascular impacts, renal damage, respiratory difficulties, and potentially Minamata sickness [7]. As a consequence, much effort has been dedicated to the development of remediation methods targeted to minimize the amount of these contaminants in wastewater, with the ultimate goal of protecting our water sources from pollution [6,8].

In wastewater treatment, various chemical and physical methods, such as coagulation, flocculation, oxidation, membrane separation, and photochemical degradation, have been thoroughly investigated to remove heavy metals [9]. Among these various approaches, adsorption has risen as the primary and preferred technique for water treatment [10,11]. This preference arises from its inherent ease of operation, resilience against harmful substances, cost effectiveness, and exceptional treatment performance. The choice of an appropriate adsorbent is of utmost importance, given the multitude of factors to consider, including but not limited to the methods of preparation, stability, selectivity, cost, and the overall availability of these adsorbents [12]. In the past few years, researchers have increasingly shown interest in harnessing intrinsically conducting polymers for various applications. These applications encompass electrochemical sensor, optical devices, bactericidal agents, and lightweight battery electrodes [13,14,15]. Among these polymers, polypyrrole stands out as a highly promising contender. It has drawn considerable interest due to its unique characteristics, including its cost-effective production, environmental stability, and remarkable electrical conductivity [16,17,18]. In the past few years, there has been an increasing fascination with investigating the creation of nanostructured PPy-based nanocomposites [19]. These composite materials are designed to increase surface areas and tackle the issues associated with irregular morphologies, aggregation and limited dispersion in water. The ultimate goal is to enhance their ability to efficiently remove pollutants from water [20]. In light of the polymerization techniques employed, the ions present within the oxidizing agent and the dimensions of the counter ions involved, it has been demonstrated that Ppy can adsorb negatively and positively charged ions [21]. However, Ppy holds substantial promise as an adsorbent material, as its adsorption capacity can be substantially improved through chemical modifications, incorporating desired functionalities to address its inherent limitations.

Integrating magnetic nanoparticles within conductive polymers has attracted significant interest as a promising solution to environmental challenges [22]. Additionally, the use of adsorption techniques encounters limitations associated with the difficulties of post-adsorption separation. Nevertheless, magnetic nanocomposites display an impressive responsiveness to external magnetic fields, simplifying their retrieval from reaction systems or wastewater streams [22]. Consequently, magnetic separation has emerged as a cost-effective, highly efficient, and expedited alternative to labor-intensive filtration [23]. Furthermore, incorporating magnetic particles into water treatment has generated substantial interest due to their outstanding physical and chemical properties [24]. However, despite notable advancements in magnetic nanoparticle synthesis, a substantial obstacle still needs to be overcome to prevent the aggregation of these magnetic particles. The primary concern revolves around the susceptibility of nanoparticles to oxidation, which becomes more pronounced as the particles decrease in size [25]. Therefore, it is crucial to establish an effective method to maintain the chemical stability of nanoparticles. One strategy for safeguarding nanoparticles involves using unique polymers like sodium alginate to create an impermeable barrier that shields the nanoparticles from oxygen exposure [26]. Sodium alginate copolymer, derived from brown seaweed, is a readily available, cost-effective, environmentally friendly, and hydrophilic biopolymer. It is currently being utilized as an adsorbent material for purifying synthetic wastewater by removing contaminants [26,27]. The incorporation of magnetic polypyrrole into alginate, a natural biopolymer, is expected to result in a composite material that can effectively address concerns related to cost, biocompatibility, and biodegradability [26,27].

This research aimed to fabricate an inexpensive adsorbent, Alg@Mag/PPy NCs, with the goal of investigating its effectiveness in removing mercury(II) from an aqueous system. Optimization of adsorption was performed by varying different factors, for instance, the Alg@Mag/PPy NCs dose, pH, temperature, and the initial mercury(II) concentration. Moreover, the isotherm, kinetic, and thermodynamic parameters were computed to elucidate the adsorption mechanism of mercury(II) onto the Alg@Mag/PPy NCs.

## 2. Experimental Section

### 2.1. Chemicals

All the chemicals utilized in this study were purchased from Sigma Aldrich (St. Louis, MO, USA). The chemicals along with their respective CAS numbers are listed as follows: Pyrrole (109-97-7), Sodium Alginate (9005-38-3), Iron(II) chloride (7758-94-3), Iron(III) chloride hexahydrate (10025-77-1), Sodium hydroxide (1310-73-2), Ammonium persulfate (APS) (7727-54-0), Hydrochloric acid (7647-01-0).

### 2.2. Synthesis of α-Fe_2_O_3_

A hydrothermal method was utilized for the fabrication of α-Fe_2_O_3_ nanoparticles. In a typical procedure, FeCl_3_·6H_2_O and FeCl_2_ with a stoichiometric ratio of Fe^3+^/Fe^2+^ equal to 2 were introduced into 60 mL of deionized water and agitated at room temperature. The pH was maintained at 9 by slowly adding NaOH solution. The mixture was then transferred into the autoclave where it was subjected to hydrothermal treatment at 180 °C for 12 h. The obtained final sample was allowed cooling to room temperature, then filtered and subjected to multiple washes with distilled water and ethanol. Finally, it was dried for 24 h in an oven set at 80 °C.

### 2.3. Synthesis of Alg@Mag/PPy NCs

**Figure 1:**
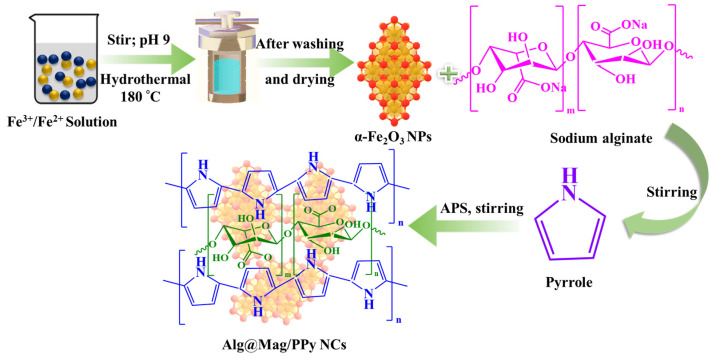
Schematic representation of the synthesis of Alg@Mag/PPy NCs.

Alg@Mag/PPy was synthesized as follows: Initially, 500 mg of α-Fe_2_O_3_ and 100 mg of sodium alginate powder were added in 100 mL of distilled water and stirred for one hour. Subsequently, 1 mM of pyrrole was introduced into the mixture and stirred at room temperature for an additional hour. Next, a 1.5 M APS was added to the reaction vessel, and the mixture was stirred for 4 h. The resulting black-color Alg@Mag/PPy NCs were collected using a magnet, washed multiple times with distilled H_2_O, followed by ethanol (C_2_H_5_OH), and dried in an oven at 50 °C. Finally, the synthesized nanocomposites were stored in an airtight vessel. Figure 1 displays the schematic illustration for synthesizing Alg@Mag/PPy NCs.

### 2.4. Adsorption Experiments

Adsorption experiments were conducted under various conditions, including mercury(II) concentration varying from 10 to 200 mg/L, temperature from 298 to 323 K, initial pH from 2 to 9, and contact time from 0 to 85 min. The adsorption processes were started by adding 30 mg of Alg@Mag/PPy NCs into the conical flasks containing 40 mL of specific concentrations (20, 40, 80 mg/L) of the mercury(II) solution. Following the establishment of equilibrium, the Alg@Mag/PPy NCs were isolated from the aqueous system utilizing magnet. Subsequently, the concentration of the remaining mercury(II) after adsorption was determined using ICP-AES. The adsorbed amount (Q_t/e_) onto the Alg@Mag/PPy NCs was determined from the change in the concentrations (C in mg/L) of mercury(II) at any time/equilibrium (t/e) by using the subsequent relation (Equation (1)) [28]:

(1) $$Q_{t/e}=\left(C_{o}-C_{t/e}\right)\times \frac{V}{m}.$$

The adsorption efficiency (%R) was determined by means of the following expression (Equation (2)):

(2) $$\%R=\frac{\left(C_{0}-C_{t/e}\right)}{C_{0}}\times 100,$$

where V is the volume of the solution (L), m is the weight of Alg@Mag/PPy NCs (g).

The linear and nonlinear equations of the P-fo (Pseudo-first-order), P-so (Pseudo-second order), and linear forms of I-D models are given in Equations (3)–(7) [29,30]. In these equations, *K*_1_ (1/min), *K*_2_ (g/min/mg), and *K*_3_ (mg/min^1/2^/g) are the rate constants of the P-fo, P-so, and I-D models, respectively, while *Q_fo_* and *Q_so_* represent the equilibrium adsorption capacities (mg/g) of P-fo and P-so models, respectively. *C* is constant [1,31].

Linear P-fo:

(3) $${ln(Q}_{e}-Q_{t})={lnQ}_{f_{o}}-K_{1}t.$$

Non-linear P-fo:

(4) $$Q_{t}=Q_{f_{o}}\times \left[1-exp\left(-K_{t}t\right)\right].$$

Linear P-so:

(5) $$\frac{t}{Q_{t}}=\frac{1}{K_{2}Q_{so}^{2}}+\frac{t}{Q_{so}}.$$

Non-linear P-so:

(6) $$Q_{t}=\frac{Q_{so}^{2}K_{2}t}{1+Q_{so}Q_{2}t}.$$

I-D:

(7) $$Q_{t}=K_{3}t^{0.5}+C.$$

Three adsorption isotherm models, specifically Langmuir, Freundlich, and Dubinin-Radushkevich, were employed to analyze the experimental data and gain theoretical insights into the adsorption process. The isotherm models are mathematically represented by Equations (8)–(15). Here, *Q_m_*, is a maximum adsorption capacity (mg/g) of Langmuir. Meanwhile, *K_L_* (L/mol) and *K_F_* (L/mg) represent the Langmuir and Freundlich model constants, respectively. These constants pertain to the adsorption capacity and the interaction between the adsorbate and adsorbent. In addition, n is a constant of Freundlich expressions which signify the intensity of adsorption. ϵ is the adsorption potential, *K_DR_* is the Dubinin–Radushkevich constant related to mean free energy of adsorption. The mean free energy of sorption can be computed using Equation (15). When the energy magnitude of E falls within the range of 8 to 16 kJ/mol, the adsorption process occurs through ion-exchange or chemisorption mechanisms. Conversely, if *E* is lower, the adsorption process is primarily of physical nature [32]. *R* (8.314 J mol/K), and *T* (298 K), are the universal gas constant, and temperature in Kelvin, respectively [1].

Linear Langmuir:

(8) $$\frac{1}{Q_{e}}=\frac{1}{Q_{m}K_{L}C_{e}}+\frac{1}{Q_{m}}.$$

Non-linear Langmuir:

(9) $$Q_{e}=\frac{Q_{m}K_{L}C_{e}}{1+K_{L}C_{e}}.$$

Linear Freundlich:

(10) $${lnQ}_{e}=\frac{1}{n}{lnC}_{e}+{lnK}_{F}.$$

Non-linear Freundlich:

(11) $$Q_{e}=K_{F}C_{e}^{\frac{1}{n}}.$$

Non-linear Dubinin–Radushkevich isotherm model:

(12) $$q_{e}=q_{m}\exp\left(-K_{DR}\epsilon^{2}\right).$$

Linear Dubinin–Radushkevich isotherm model:

(13) $$lnq_{e}=lnq_{m}-K_{DR}\epsilon^{2}.$$

(14) $$\epsilon =RTln\left(1+\frac{1}{C_{e}}\right).$$

(15) $$E=\frac{1}{\sqrt{2K_{DR}}}.$$

In these isotherms, the Freundlich and the Langmuir models are the multilayer sorption on the heterogeneous surface and the monolayer sorption on the uniform surface, respectively. The Dubinin–Radushkevich isotherm model serves as a valuable tool for determining whether the sorption process is of a chemical or physical nature, and it aids in the assessment of the average energy of sorption [32].

The thermodynamic parameters, i.e., Gibb’s free energy (Δ*G*), enthalpy (Δ*H*), and entropy (Δ*S*) are computed by the Equations (16) and (17) [1].

(16) $$\Delta G=-RTlnK_{c},$$

(17) $${lnK}_{c}=\frac{-\Delta G}{RT}=\frac{\Delta S}{R}-\frac{\Delta H}{RT},$$

where *K_c_* is the thermodynamic constant and can be estimated by the ratio of adsorption capacity to the residual concentration of metal ion molecule in the solution.

### 2.5. Instrumentation Details

Surface morphology was observed by using Scanning Electron Microscope (SEM, JEOL, JSM6510LV, Tokyo, Japan) and Transmission Electron Microscope (TEM, JEOL, 2100). The crystallinity of the nanocomposite was deliberated by X-ray Powder Diffraction (MiniFlex™ II benchtop XRD system, Rigaku Corporation, Tokyo, Japan) in the 2θ range of 10–80° at 40 kV and a current of 30 mA. The FTIR spectra spanning a range of 4000 to 400 cm^−1^ were captured to examine functional group compositions, utilizing the Nicolet IS50 Fourier-transform infrared (FTIR) spectrometer from Thermo Fisher Scientific (Waltham, MA, USA). Magnetic characteristics were assessed under ambient conditions, employing a vibrating sample magnetometer with a peak magnetic field strength of 20 kOe (VSM) (Lakeshore, 7410).

## 3. Results and Discussion

### 3.1. Characterization

**Figure 2:**
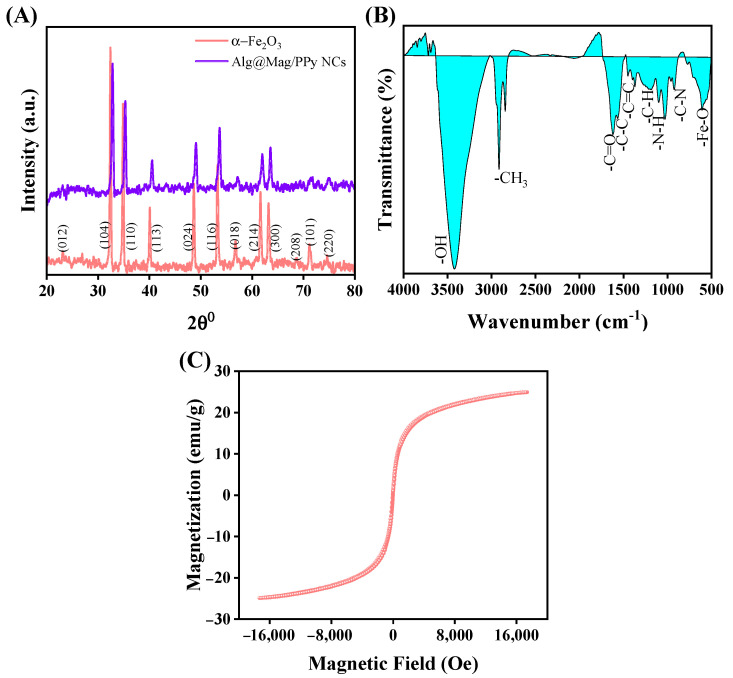
(**A**) XRD of α-Fe_2_O_3_ and Alg@Mag/PPy NCs, (**B**) FTIR of Alg@Mag/PPy NCs (**C**) VSM of Alg@Mag/PPy NCs.

Figure 2A presents the X-ray diffraction (XRD) pattern of the synthesized α-Fe_2_O_3_ and Alg@Mag/PPy NCs. In the XRD pattern of the prepared α-Fe_2_O_3_ nanoparticles, the peaks are observed at 23.15, 32.45, 34.87, 40.08, 48.87, 53.35, 56.79, 61.71, 63.21, 68.73, 71.15, and 74.71° corresponding to the (012), (104), (110), (113), (024), (116), (018), (214), (300), (208), (101) and (220) planes, respectively. These peaks serve as strong evidence for the formation of the α-Fe_2_O_3_ phase, as they align with the standard data from the JCPDS Card No. 33-0664. Using Scherrer’s equation [33], the crystallite size of α-Fe_2_O_3_ is found to be 12.05 nm. This result is confirmatory with TEM micrographs [34]. Notably, no impurity phases are detected, affirming the sample’s purity as a hexagonal phase α-Fe_2_O_3_ crystalline structure [35]. In the XRD pattern of Alg@Mag/PPy NCs, all the α-Fe_2_O_3_ peaks remain discernible, albeit with a slight shift towards higher 2θ values. This slight shift may be due to the interactions between the α-Fe_2_O_3_ phase, and other components in the NCs, such as Alg and PPy, can affect the crystal lattice of α-Fe_2_O_3_, leading to peak shifts. The interaction between α-Fe_2_O_3_ and Alg or PPy can introduce strain into the α-Fe_2_O_3_ lattice. Additionally, a broad hump, centered in the range of 2θ = 20 to 30°, was observed. This hump corresponds to the diffraction planes of PPy and Alg, indicating that the desired NCs were successfully formed [34,36,37].

In accordance with the Fourier-Transform Infrared (FTIR) spectrum presented in Figure S1, Supplementary File, and Figure 2B for Alg, α-Fe_2_O_3_, and Alg@Mag/PPy NCs, respectively, several characteristic vibrational modes and peaks can be discerned. In Supplementary File, as depicted in Figure S1, the extensive spectral bands within the 2600–3600 cm^−1^ range can be ascribed to the stretching vibrations of -OH bonds present in Alg and adsorbed water on the nanoparticles. The Alg spectrum exhibits a faint peak at 2926 cm^−1^, associated with–CH_2_ groups. Furthermore, characteristic Alg bands are discernible at 1628 and 1407 cm^−1^, representing the asymmetric and symmetric stretching vibrations of the carboxylate, respectively. Additionally, the bands at 1114 and 1031 cm^−1^ indicate the presence of C-O bonds [38]. The bands at 527 cm^−1^ observed on the α-Fe_2_O_3_ spectrum are the signature of the Fe-O bonds [39]. In the FTIR spectrum of Alg@Mag/PPy NCs (Figure 2B), the peak at approximately 582 cm^−1^ indicates the stretching vibrations associated with the Fe-O bonds, as reported in reference [40]. Furthermore, the FTIR spectrum reveals distinctive peaks corresponding to various vibrational modes within the molecular structure of the material. Specifically, the C-C and C=C in-plane bending vibrations of the pyrrole rings are observed at approximately 1554 cm^−1^ and 1439 cm^−1^, respectively. Additionally, a peak at 910 cm^−1^ is attributed to the C-N stretching vibration. The N-H vibrations manifest at 1086 cm^−1^, while the C-H in-plane vibrations are represented by a peak at 1175 cm^−1^, as reported in references [41,42]. The characteristic peaks of the alginate are the peaks at 1629 cm^−1^ (C=O bond) and 1408 cm^−1^ [43]. Furthermore, the FTIR spectrum indicates absorption peaks at 3418 cm^−1^ is the stretching vibration of -OH and the peaks at 2924 and 2860 cm^−1^ represent the symmetric and asymmetric stretching vibration of CH of carbon sp^3^ [25,44].

The X-ray Photoelectron Spectroscopy (XPS) data provide valuable insights into the chemical composition and formation of Alg@Mag/PPy NCs. The XPS spectra reveal the existence of C1s, N1s, Fe2p, and O1s, confirming the successful creation of Alg@Mag/PPy NCs. In the C 1s XPS spectrum of Alg@Mag/PPy NCs, as depicted in Figure S2A, Supplementary File, peaks at 287.03, 285.34, 284.52, and 283.38 eV are allocated to the C=N or C-O, C–N, and C=C bonds, respectively, as reported in literature [45]. Additionally, a detailed examination of the N 1s spectrum (Figure S2B, Supplementary File) reveals peaks centered at 398.27 and 394.35 eV, corresponding to C-N and C=N, respectively, in Alg@Mag/PPy NCs [46]. The O 1s XPS spectrum (Figure S2C Supplementary File) is divided into four distinct parts, with peak positions at 530.37 eV (Fe–O), 531.92 eV (C–O or C-O-Fe), and 532.56 eV (Fe–OH or C=O) in Alg@Mag/PPy NCs [47,48,49]. The fitted Fe2p spectrum in Figure S2D, Supplementary File, exhibits two wide-ranging peaks at 723.11 and 710.01 eV, corresponding to Fe 2p1/2 and Fe 2p3/2, respectively [47]. To delve further into the adsorption mechanisms, the XPS spectra of Alg@Mag/PPy NCs after Hg(II) ions adsorption are examined. The analysis post adsorption reveals the presence of mercury, confirming the successful adsorption process. Two discernible peaks at 100.29 eV and 104.04 eV (as seen in Figure S2E, Supplementary File) belong to Hg 4f7/2 and Hg 4f5/2, respectively [50]. Moreover, C1s, N1s, O1s and Fe 2p spectra, as given in Figure S2A–D, Supplementary File, respectively, exhibit notable changes upon the adsorption of Hg(II). These changes are manifested as shifts in peak positions and collectively indicate the successful adsorption of Hg(II) onto the Alg@Mag/PPy NCs. The adsorption mechanism of Hg(II) onto the Alg@Mag/PPy NCs primarily encompasses surface complexation, electrostatic attraction, and chemical interactions, as illustrated in Section 3.2.

The magnetic properties of Alg@Mag/PPy NCs are comprehensively investigated using VSM. The magnetic curve in Figure 2C, showcases the typical magnetic behavior of Alg@Mag/PPy NCs, highlighting their inherent magnetism. Notably, the saturation magnetization of Alg@Mag/PPy NCs is 23.657 emu/g. This substantial magnetization underscores the composite’s magnetic character, making it eminently suitable for conventional magnetic separation techniques. Furthermore, the intrinsic coercivity (the point on the hysteresis loop where the magnetization reaches zero; we note the corresponding magnetic field strength) and retentivity (the point on the hysteresis loop where the magnetization is maximum when the applied magnetic field is zero) of Alg@Mag/PPy NCs are determined to be 32.487 Oe and 1.564 emu/g, respectively, through VSM analysis. These values are indicative of the NCs’ robust magnetic attributes. Notably, the magnetic hysteresis loop exhibited by Alg@Mag/PPy NCs, as depicted in Figure 2C, adopts an S-shaped configuration and low retentivity. This distinct behavior indicates excellent dispersibility and facile demagnetization characteristics. In light of these findings, Alg@Mag/PPy NCs emerge as promising contenders for applications in wastewater treatment. Their magnetic nature enables efficient isolation from water by simply employing a magnetic field. This remarkable property enhances their utility in environmental cleanup efforts and underscores their potential importance in addressing water pollution challenges [51].

**Figure 3:**
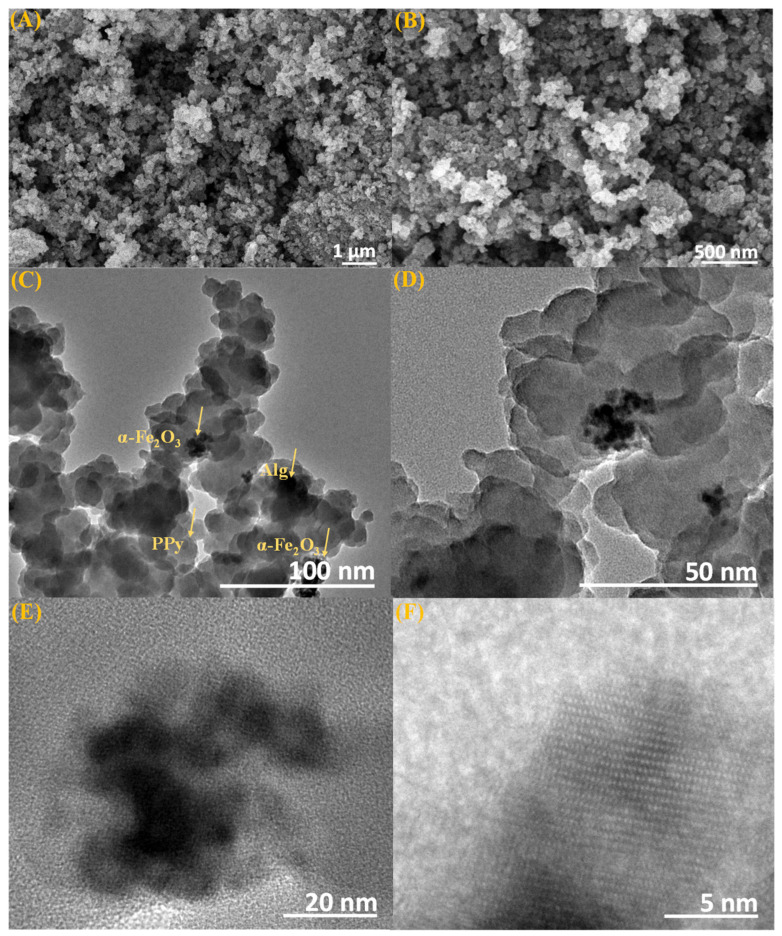
SEM images of Alg@Mag/PPy NCs at (**A**) 1 μm, (**B**) 500 nm; TEM images of Alg@Mag/PPy NCs at (**C**) 100 nm, (**D**) 50 nm, (**E**) 20 nm, (**F**) 5 nm.

The scanning electron microscope (SEM) images, as depicted in Figure 3A,B, vividly illustrate the structural characteristics of the Alg@Mag/PPy NCs. These images reveal the presence of agglomerated spherical-shaped particles composed of α-Fe_2_O_3_, alongside the deposition of rugged and furry polypyrrole (PPy) particles. Notably, the images also showcase a discernible light-contrast matrix of Alg, further accentuating the intricate architecture of Alg@Mag/PPy NCs.

The transmission electron microscopy (TEM) images presented in Figure S2, Supplementary file, and Figure 3C–F offer valuable insights into the structural characteristics of α-Fe_2_O_3_ and Alg@Mag/PPy nanocomposites, respectively. The synthesized α-Fe_2_O_3_ nanoparticles exhibit a consistent spherical morphology, with sizes varying between 8 and 13 nm, Figure S2, Supplementary File. Figure 3C–F illustrate distinct features within the Alg@Mag/PPy nanocomposites, revealing an intricate interplay of materials. Notably, the TEM images distinctly capture the diffused morphology of the Ppy particles, which are dispersed throughout the composite matrix. This diffused distribution underscores the complex and finely tuned interconnection of Ppy within the Alg@Mag/PPy nanocomposites. Moreover, the TEM images also unveil the presence of dark regions that sharply contrast with the presence of Alg, and also provide evidence of the successful incorporation of agglomerated minute particles which correspond to α-Fe_2_O_3_ into the nanocomposite structure. The nitrogen adsorption–desorption isotherms for Alg@Mag/PPy, as illustrated in Figure S2, Supplementary File, indicate a type-IV isotherm profile, with the BET surface area of Alg@Mag/PPy determined to be 49.834 m^2^/g.

### 3.2. Adsorption Study

**Figure 4:**
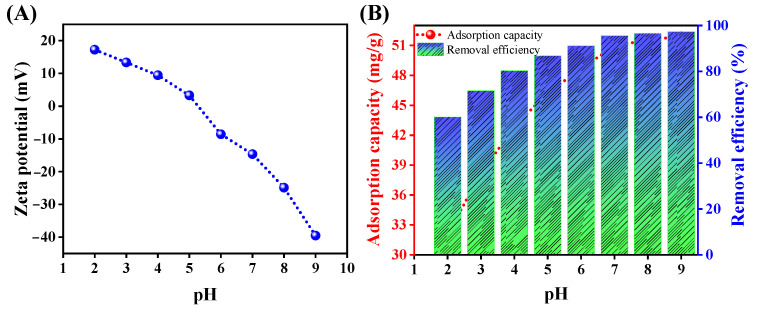
(**A**) Zeta potential of the Alg@Mag/PPy NCs at different pH. (**B**) Influence of pH on the adsorption capacity and removal efficiency of mercury(II) onto the Alg@Mag/PPy NCs (Experimental conditions: Adsorbent dose = 0.03 g/40 mL, Time = 60 min, Concentration = 40 mg/L, Temperature = 303 K).

**Figure 5:**
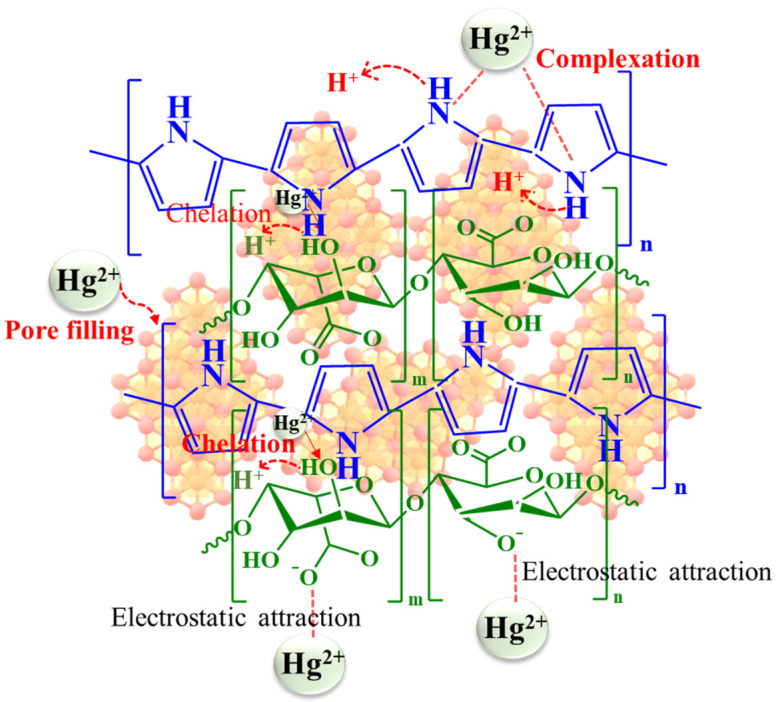
Mechanism for the removal of mercury(II) via Alg@Mag/PPy NCs.

The charge on the adsorbent surface, the ionic statuses of oxygen-comprising functional groups, and the ionic forms of metal can be greatly influenced by variations in pH. The influence of pH was studied across a pH range from two to nine. As depicted in Figure 4B, the adsorption efficiency of the Alg@Mag/PPy NCs was notably affected by the pH solution. Specifically, the adsorption capacity for mercury(II) increased as the pH level increased. Nonetheless, magnetized adsorbents often exhibit the drawback of iron leaching when exposed to low pH conditions. The leaching experiments were conducted across a spectrum of pH values between two and nine and unveiled that the presence of leached iron in the solution was solely detected at a pH of two. No leaching was observed at pH levels beyond three [52,53]. Consequently, it was observed that, at a pH of two, the adsorption of mercury was significantly lesser. Furthermore, zeta potentials serve as a common method for assessing the electric charges present on particle surfaces in aqueous solutions. The point at which the Alg@Mag/PPy NCs exhibit zero charge, known as the point of zero charge (pH_PZC_), was established by gauging the zeta potential of these nanocomposites at varying pH levels. As displayed in Figure 4A, the zeta potentials of Alg@Mag/PPy NCs exhibit a strong negative charge, and this negative charge intensity increases as the pH level rises from 6.0 to 9.0. The point at which the charge neutrality is achieved (known as pH_PZC_) for Alg@Mag/PPy NCs was determined to be approximately 5.31. This finding suggests that when the pH is lower than the pH_PZC_, Alg@Mag/PPy NCs have a positively charged surface, making them capable of attracting anions. Conversely, when the pH exceeds the pH_PZC_, the surface charge of Alg@Mag/PPy NCs becomes negative, which can be advantageous for the sorption of cations. When the pH level was increased from two to seven, there was a rapid enhancement in the adsorption capability of the Alg@Mag/PPy nanocomposites, ultimately reaching 95.58% (50 mg/g) at a pH of 7.0. As the solution pH values continued to increase beyond seven, the adsorption efficiency of the Alg@Mag/PPy nanocomposites kept growing, albeit at a progressively slower pace. The observed phenomenon can be attributed to the presence of various forms of mercury(II) in water. The types of mercury compounds found in water vary depending on the pH of the solution. Specifically, at a pH below 4.0, the predominant species are Hg^2+^, with a smaller presence of HgCl^+^. In the pH value range of four to six, the main species shift to HgCl_2_ with a trace amount of Hg(OH)_2_. As the pH level increases from six to eight, the species include Hg(OH)^3+^, HgOH^+^, HgCl_2_, and a small quantity of Hg(OH)_2_. Ultimately, when the pH level exceeds eight, Hg(OH)_2_ predominantly precipitates as an insoluble compound [54,55,56,57]. Conversely, at lower pH values, an electrostatic repulsion occurs between Alg@Mag/PPy NCs and Hg^2+^, impeding the efficient elimination of mercury(II). As the pH value escalates within the range of five to seven, the surface of Alg@Mag/PPy NCs becomes negatively charged, thereby augmenting their capacity to adsorb both Hg^2+^ and Hg(OH)_2_. Nonetheless, once the solution’s pH level exceeded seven, the rate of increase in adsorption capacity started to diminish gradually. This slowdown can be ascribed to the formation of Hg(OH)_2_ [55]. At the same time, it is possible that this phenomenon stemmed from the nitrogen atoms’ lone electron pairs, which interacted with Hg^2+^ to create relatively stable complexes, thereby facilitating the elimination of mercury(II) [55,58]. Figure 5 shows the possible interaction between mercury(II) and Alg@Mag/PPy NCs. Furthermore, elevating the pH level led to the generation of active sites with negative charges through the deprotonation of oxygen-rich functional groups and the PPy. Consequently, a pH level of seven was chosen for the next set of adsorption experiments. Moreover, it was also observed that after adsorption, the pH was decreased. It can be concluded from these trends that as more metal ions are adsorbed onto the Alg@Mag/PPy NCs, more hydrogen ions are released into the solution. The hydrogen ion sources are most likely the carboxylic and hydroxyl groups of the Alg@Mag/PPy NCs. These groups are generally considered responsible for cation exchange capacity of Alg@Mag/PPy NCs [59,60].

**Figure 6:**
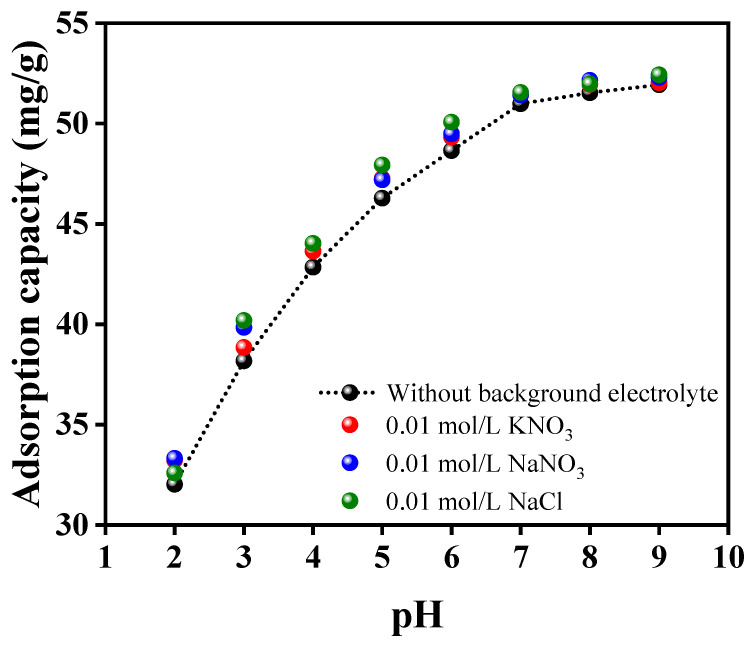
Effect of 0.01 mol/L background electrolyte on mercury(II) removal by Alg@Mag/PPy NCs.

To explore the impact of background electrolyte on the adsorption of mercury(II), the experiments were conducted by examining the adsorption of mercury(II) onto Alg@Mag/PPy NCs across a range of pH values in solutions containing 0.01 mol/L of NaNO_3_, KNO_3_ and NaCl. As illustrated in Figure 6, the inclusion of NaNO_3_, KNO_3_ and NaCl yielded a minor improvement in the removal of mercury(II) across a range of pH levels. Drawing from the preceding discussion, it becomes evident that mercury(II) ions have the potential to establish inner-sphere complexes with the surfaces of Alg@Mag/PPy nanocomposites. Nevertheless, it is worth noting that background electrolyte ions typically occupy the same plane as the outer-sphere complexes [61]. Hence, the influence of NaNO_3_, KNO_3_ and NaCl on the adsorption of mercury(II) was found to be relatively modest. The primary factor behind the improved mercury(II) adsorption is the mitigation of electrostatic repulsion between mercury(II) ions and the Alg@Mag/PPy NCs, facilitated by the presence of background electrolytes [62].

**Figure 7:**
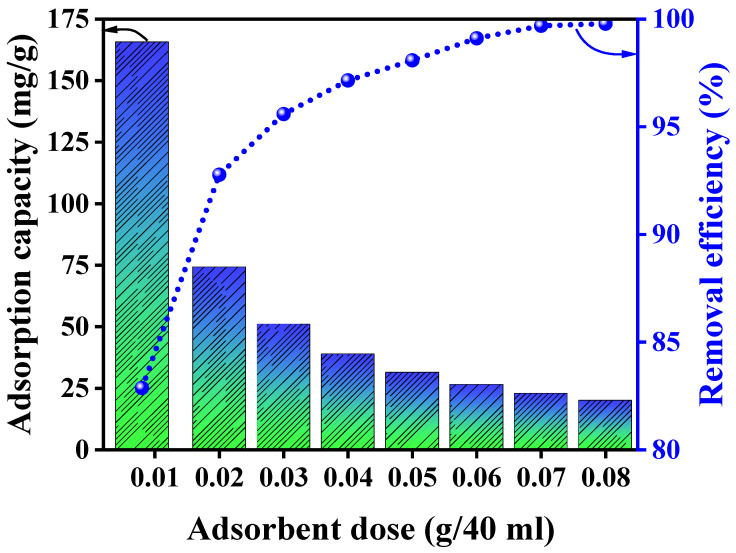
Influence of Alg@Mag/PPy NC dosage on the adsorption capacity and removal efficiency of mercury(II) (Experimental conditions: pH = 7, Adsorbent dose = 0.03 g/40 mL, Time = 60 min, Concentration = 40 mg/L, Temperature = 303 K).

A series of experiments were conducted to investigate the impact of varying the amount of adsorbent used on the adsorption of mercury(II) ions, as presented in Figure 7. The outcomes demonstrate that as the quantity of adsorbent increased from 8 to 80 mg/40 mL within a working volume of 40 mL, the percentage of mercury(II) removal experienced a notable increase, rising from 82.88% to 99.78%. However, the equilibrium adsorption capacity, which denotes the quantity of metal adsorbed per unit of adsorbent, decreased from 165.75 mg/g to 19.96 mg/g. The decrease in adsorption capacity can be reasonably ascribed to the concentration gradient between the adsorbent and adsorbate. As the level of adsorbent concentration increases, the amount of adsorbate captured per unit of adsorbent mass diminishes [63,64]. Conversely, the enhancement in removal efficiency with increasing Alg@Mag/PPy NC dosage is likely due to NCs’ well-modified surface properties, substantial surface area, and the presence of easily accessible functional groups and active sites for adsorption. However, it is worth noting that when the adsorbate becomes limited within the system, a further rise in the dose of Alg@Mag/PPy NCs up to approximately 0.03 g/40 mL has only a marginal effect on removal efficiency. The optimal dose for achieving the highest sorption of mercury(II) onto the Alg@Mag/PPy NCs was determined to be 0.03 g/40 mL, resulting in a maximum sorption capacity (removal efficiency) of 50.98 mg/g (95.59%). As a result, the most effective quantity for achieving the highest mercury(II) sorption onto the Alg@Mag/PPy NCs was determined to be 0.03 g/40 mL.

**Figure 8:**
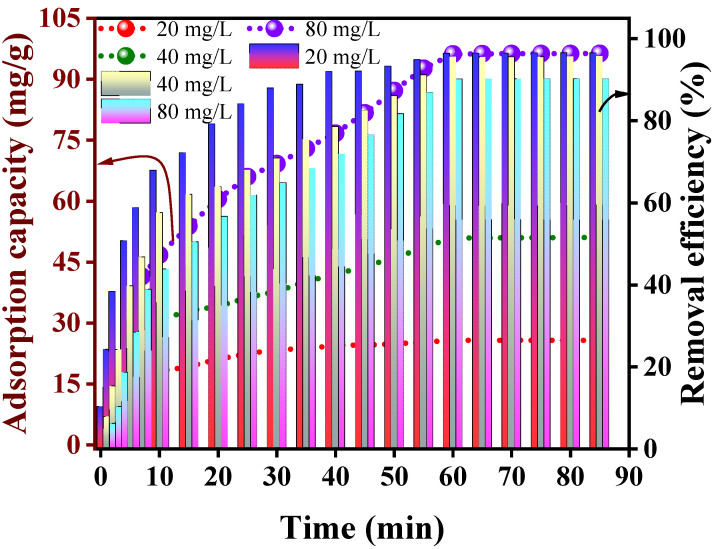
Influence of interaction time on the adsorption capacity and removal efficiency of mercury(II) onto the Alg@Mag/PPy NCs (Experimental conditions: Adsorbent dose = 0.03 g/40 mL, pH = 7, Concentration = 40 mg/L, Temperature = 303 K).

The experimental findings about the impact of reaction duration can be observed in Figure 8. According to the data presented in Figure 8, there was a swift rise in the adsorption capacity of the Alg@Mag/PPy NCs as the contact time increased to 10 min. Afterward, the rate of this increase in adsorption began to slow down, eventually reaching a state of equilibrium around 60 min. Accordingly, the maximum adsorption capacity (removal efficiency) of the Alg@Mag/PPy NCs at concentrations of 20, 40, and 80 mg/L was found to be 25.72 mg/g (96.45%), 50.98 mg/g (95.58%), and 96.19 mg/g (90.18%), respectively, when equilibrium was attained (60 min). These outcomes can be ascribed to the presence of numerous metal binding sites on the Alg@Mag/PPy NCs’ surface initially. Over time, the quantity of accessible adsorption points decreased, and the repelling interactions between the mercury(II) ions present on the Alg@Mag/PPy NCs surface and those within the solution intensified. This, in turn, made it increasingly challenging for mercury(II) ions to occupy the limited remaining active spots. Furthermore, the discrepancy in mercury(II) concentrations between the solid surface and the liquid phase slowly declined, causing a reduction in the rate of adsorption. The optimum adsorption at all concentrations of mercury(II) was attained within 60 min of interaction time.

**Figure 9:**
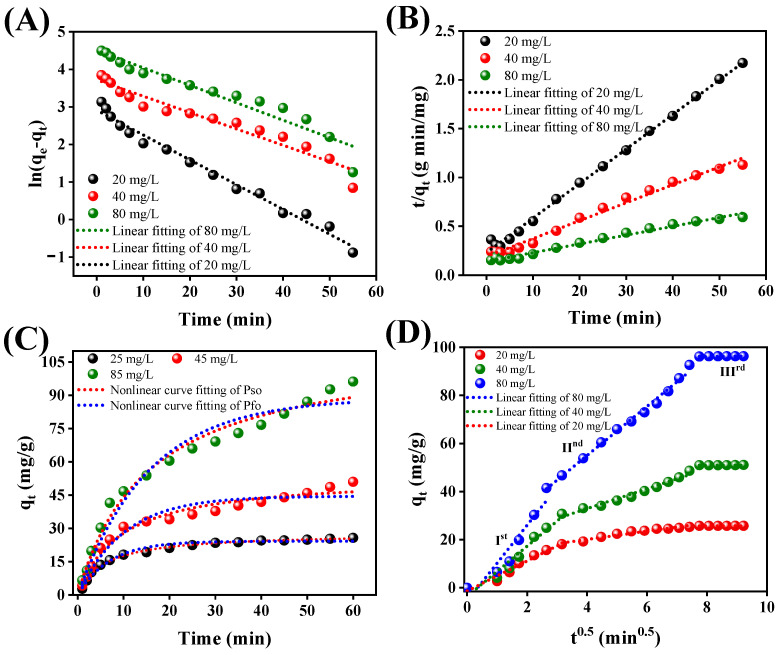
(**A**) Linear fitting curves of P-fo kinetic models for mercury(II) using Alg@Mag/PPy NCs; (**B**) Linear fitting curves of P-so kinetic models for mercury(II) using Alg@Mag/PPy NCs; (**C**) Nonlinear fitting curves of P-fo and P-so kinetic models for mercury(II) using Alg@Mag/PPy NCs; (**D**) Three-stage linear fitting of I-D for mercury(II) adsorption onto the Alg@Mag/PPy NCs (Ist, IInd, and IIIrd are the surface-diffusion, pore-diffusion, and equilibrium approaching stage) (Experimental conditions: pH = 7, Adsorbent dose = 0.03 g/40 mL, Temperature = 303 K).

Adsorption, a physicochemical phenomenon, includes the movement of adsorbate substances from a solution phase to the adsorbent surface. Various kinetic models are employed to examine the transfer characteristics of adsorbed molecules. Kinetic study plays a pivotal role when assessing adsorption as a cohesive process. Evaluating the equilibrium data through kinetic analysis allows us the possibility to ascertain the time needed for metallic adsorption [65]. The adsorption kinetics essentially govern the duration required to achieve equilibrium and profoundly influence the mechanism of adsorption [66]. To conclude our investigations into the kinetics that govern this process, we analyzed the obtained time-dependent adsorption data using both linear and nonlinear models, specifically the Pfo and Pso models (Equations (3)–(6)) as shown in Figure 9A–C. The associated parameters for these models, along with their respective correlation coefficients, can be found in Table 1. The Pfo equation examines the distribution of occupied and vacant adsorption sites and characterizes adsorption within solid-solution systems by considering the solid’s adsorption capacity. On the other hand, the Pso equation serves as a tool for examining chemical kinetics in liquid solutions. It highlights that chemisorption is crucial as the rate-determining step in surface adsorption [67]. This model ascribes the process of adsorption to the physicochemical interactions occurring between the solid and solution phases [54]. The experimental findings demonstrated a notably low R^2^ value for mercury(II) ions in the Pfo model. In contrast, the compatibility between the experimental data and the Pso model was quite favorable, yielding a higher R^2^ value. Consequently, the Pso model offered a more coherent explanation for the experimental data. Additionally, the calculated equilibrium capacity (q_e_,_cal_) derived from the Pso equation closely approximated the experimental value (q_e_,_exp_). This observation underscores the accurate fit of the experimental adsorption data for mercury(II) on the Alg@Mag/PPy NCs to the Pso equation. Furthermore, as indicated in Table 1, it is evident that the rate constants (K_2_) for Pso exhibit a decreasing trend with the rise in mercury(II) concentration. This phenomenon can be attributed to the insufficient number of adsorption sites available to capture mercury(II) at elevated concentrations, at the same amount of the adsorbent dose [53,54].

Figure 9D illustrates the intra-particle diffusion plots concerning the adsorption of metal ions onto Alg@Mag/PPy NCs. Notably, the plot exhibits non-linearity across the entire time range, suggesting that multiple factors influenced mercury adsorption (II). In Figure 9D, it is evident that the plots display a clear tri-linearity, indicating the presence of three distinct stages in the sorption process. Furthermore, the intra-particle diffusion rate constants calculated from Equation (7) for each of these stages follow the sequence of k_I_ > k_II_ > k_III_, as depicted in Table 1 [1]. This phenomenon can be elucidated as follows: the initial segment (Ist stage) characterized by a steep slope corresponds to surface diffusion, during which mercury(II) ions are rapidly adsorbed by the active groups on the surface of Alg@Mag/PPy. The subsequent stage (IInd stage), with a less pronounced slope, indicates the penetration of metal ions into the pores of Alg@Mag/PPy. During this phase, mercury(II) ions are adsorbed by the inner surfaces of these pores. The final portion (IIIrd stage), the equilibrium phase, is marked by diffusion rate constants approaching zero, indicating that the adsorption process reached equilibrium.

Figure S1A,B, Supplementary File, exhibit the impact of varying mercury concentrations (II) on the equilibrium capacity and removal percentage of Alg@Mag/PPy NCs adsorbent under optimal conditions. As depicted in Figure S1B, Supplementary File, the highest mercury(II) removal percentage occurred at a concentration of 10 mg/L, while the lowest was observed at 200 mg/L of mercury(II) ions. Notably, an increase in mercury(II) concentration resulted in an augmented adsorption capacity (Figure S1A, Supplementary File), likely owing to the heightened driving force created by the concentration gradient. Conversely, at high mercury(II) concentrations, active sites may become saturated, leading to a reduction in removal percentage [67]. Furthermore, it is plausible that as metallic ion concentration rises, a strong driving force between the mercury(II) ions may impede their adsorption by Alg@Mag/PPy NCs [68].

**Figure 10:**
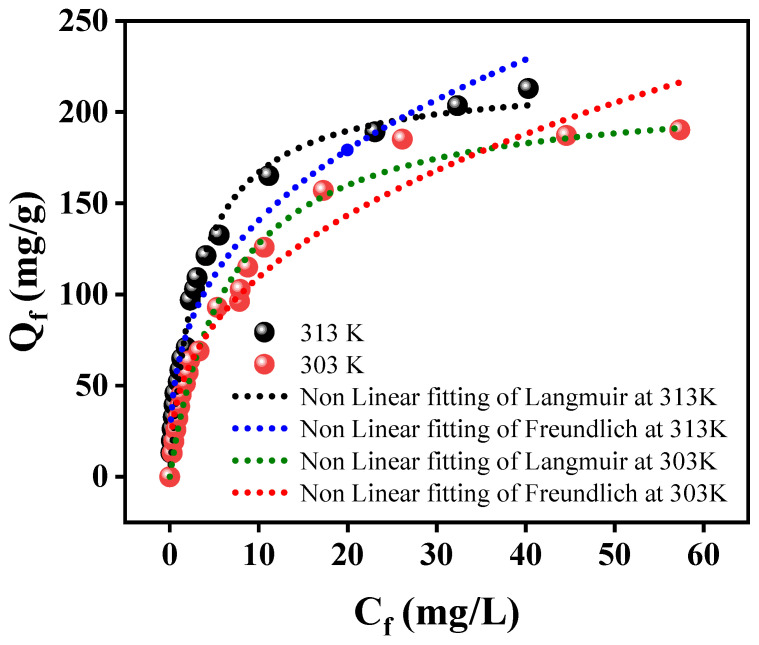
Non-linear fitting curves of Langmuir, and Freundlich model for mercury(II) adsorption onto the Alg@Mag/PPy NCs (Experimental conditions: pH = 7, Adsorbent dose = 0.03 g/40 mL, Time = 60 min).

The study of adsorption isotherms is a crucial approach for assessing the effectiveness of an adsorbent. It enables the determination of the adsorbent’s maximum capacity for adsorption, as well as provides insights into the nature of the interaction mechanisms between the adsorbent and the adsorbate. The experimental data were fitted using two adsorption isotherms models such as Langmuir, and Freundlich models. Linear and non-linear forms of these isotherm models are given in Equations (8) and (11), respectively. The non-linear fitting adsorption isotherm plots of mercury(II) are presented in Figure 10. The calculated isotherm parameters are presented in Table 2. Based on the R^2^ values, it appears that the Langmuir model provides the most suitable fit for removing mercury(II) onto Alg@Mag/PPy NCs. The feasibility of mercury(II) removal by Alg@Mag/PPy was substantiated by a dimensionless parameter known as a separation factor (R_L_${=\frac{1}{1+K_{L}C}}_{o}$). The R_L_ values obtained serve as an indicator of the adsorption process, classifying whether it is irreversible (R_L_ = 0), favorable (0 < R_L_ < 1), unfavorable (R_L_ > 1), or linear (R_L_ = 1) [53]. Across various initial adsorbate concentrations, the R_L_ values fell within the range of 0 to 1, demonstrating the favorable nature of the adsorption process. According to the Langmuir isotherm model, the maximum mercury(II) sorption capacity onto Alg@Mag/PPy NCs was estimated to be 164.24 mg/g at 303 K and 169.42 mg/g at 313 K. The rising adsorption capacity value with temperature suggests that higher temperatures promote the adsorption of mercury(II), which may be due to the increase in the kinetic energy and the mobility of the mercury(II) ions to be adsorbed on the Alg@Mag/PPy NCs [69,70]. Moreover, higher temperatures often lead to a reduction in the viscosity of the solution, which enhances mass transfer rates and promotes the efficient transport of adsorbate molecules from the bulk solution to the adsorbent surface [69,71]. This phenomenon can contribute to the increased adsorption capacity observed with rising temperatures. An elevated adsorption capacity indicates effective mercury(II) coverage on the surface of Alg@Mag/PPy NCs. This phenomenon can be attributed to the abundant negatively charged adsorption sites created by the presence of polypyrrole, alginate, and iron oxide collectively. Furthermore, the growing correlation between K_L_ and temperature suggests that the affinity of mercury(II) for Alg@Mag/PPy NCs strengthens as the temperature rises [53,72,73]. The values obtained from the Langmuir isotherm model in the adsorption of mercury(II) onto the Alg@Mag/PPy NCs was closely aligned with the data reported in the literature [55,74,75,76,77,78,79,80] given in Table 3. In order to ascertain whether the adsorption process is of a physical or chemical nature, we analyzed the equilibrium adsorption data at varying temperatures by fitting it to the Dubinin–Radushkevich isotherm model (as shown in Figure S2 of Supplementary File). According to existing literature, the sorption energy typically falls within the range of 8 to 16 kJ/mol for chemical or ion exchange processes, while it remains below 8 kJ/mol for physical adsorption [32]. Consequently, in the case of mercury(II) adsorption, it can be deduced that the process primarily involves physical adsorption, taking place between Alg@Mag/PPy NCs and mercury(II).

**Figure 11:**
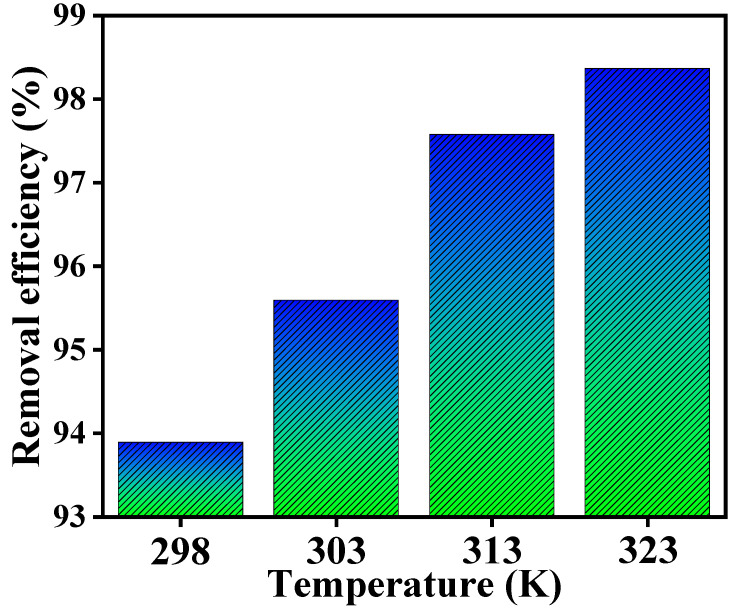
Influence of temperature on the adsorption capacity and removal efficiency of mercury(II) using Alg@Mag/PPy NCs (Experimental conditions: pH = 7, Time = 60 min, Concentration = 40 mg/L, Adsorbent dose = 0.03 g/40 mL).

Figure 11 illustrates that as the temperature rises, the adsorption capacity of the adsorbent also increases. Table 4 presents the calculated thermodynamic factors for the removal of mercury(II) onto the Alg@Mag/PPy NCs calculated by using Equations (16) and (17). The data in Table 4 reveal that the adsorption of mercury(II) exhibits ΔG° values of less than zero, confirming that the adsorption process is spontaneous. The positive ΔH° value (43.123 kJ/mol) indicates an endothermic phenomenon. This suggests that elevating the temperature within a specific range is advantageous for the adsorption process. The underlying rationale is conjectured to be the accelerated motion of metal ions as the temperature rises. This heightened motion fosters collisions between the adsorbent and adsorbate, thereby enhancing ion exchange during the adsorption process. Based on Table 4, the ΔS° value was greater than zero, which indicates an increased degree of freedom at the solid–liquid interface during adsorption. This heightened freedom ultimately results in a decrease in entropy. Conversely, when bound water is introduced to initially hydrated metal ions within the solvent system, it enhances their degree of freedom, leading to an elevation in entropy. During this procedure, the rise in entropy resulting from the release of water molecules outweighed the decrease in entropy caused by the uptake of metal ions, leading to an overall rise in the system’s degree of freedom (ΔH° > 0). To sum it up, the adsorption of mercury(II) by Alg@Mag/PPy NCs was demonstrated as a spontaneous and endothermic reaction.

## 4. Conclusions

The current research investigation demonstrates the remarkable efficacy of Alg@Mag/PPy NCs as an adsorbent for the confiscation of mercury(II) from synthetic wastewater. Various parameters, including the dosage of Alg@Mag/PPy NCs, the interaction time between mercury(II) and Alg@Mag/PPy NCs, the initial concentration of mercury(II), solution pH, and system temperature, exert considerable influence on the adsorption of mercury(II). The optimal pH level for mercury(II) adsorption is identified as seven. The analysis of the isotherms confirms that the Langmuir model is the most suitable for describing the equilibrium data of mercury(II), yielding a maximum monolayer sorption capacity of 213.72 mg/g at 303 K. Furthermore, the time-dependent experimental data confirm the applicability of the Pso kinetic model, with adsorption equilibrium achieved within 60 min. In conclusion, Alg@Mag/PPy NCs emerge as a highly efficient adsorbent offering easy magnetic separation capabilities, making them a capable candidate for confiscating mercury(II) from an aqueous system.

## Figures and Tables

**Table 1:** Kinetic factors for the removal of mercury(II) onto the Alg@Mag/PPy NCs.

Kinetics	Parameters	20 mg/L	40 mg/L	80 mg/L	Kinetics	Parameters	20 mg/L	40 mg/L	80 mg/L
P-fo	Q_exp_ (mg/g)	25.7209	50.9800	96.1867	I-D_I_ *	K_I_ (mg/min^1/2^/g)	6.2558	10.4559	15.7822
	Q_fo_ (mg/g)	18.368	41.264	90.477		C_I_	−1.3585	−3.6302	−5.5511
	K_1_ (1/min)	0.066	0.0435	0.0465		R_adj_^2^	0.9597	0.9439	0.8868
	R_adj_^2^	0.9827	0.9368	0.9218		R^2^-linear	0.9664	0.9532	0.9094
	R^2^-linear	0.9839	0.9413	0.9274	I-D_II_ *	K_II_ (mg/min^1/2^/g)	1.7178	4.1280	10.2239
						C_II_	13.220	16.3937	14.1056
P-so	Q_exp_ (mg/g)	25.7209	50.9800	96.1867		R_adj_^2^	0.9428	0.9702	0.9925
	Q_so_ (mg/g)	28.249	54.348	111.111		R^2^-linear	0.9491	0.9736	0.9933
	K_2_ (g/min/mg)	5.48 × 10^−3^	1.78 × 10^−3^	5.82 × 10^−5^	I-D_III_ *	K_III_ (mg/min^1/2^/g)	0.0277	0.0628	0.0731
	R_adj_^2^	0.9972	0.9869	0.9822		C_III_	25.5047	50.4595	95.9451
	R^2^-linear	0.9974	0.9869	0.9834		R_adj_^2^	0.9428	0.5587	0.8075
						R^2^-linear	0.9524	0.6470	0.8460

* I-D_I_, I-D_II_, and I-D_III_ are the three-stage linear fitting of I-D model for mercury(II) adsorption onto the Alg@Mag/PPy NCs given in Figure 9D.

**Table 2:** Nonlinear Isotherm models values for the removal of mercury(II) via Alg@Mag/PPy NCs.

Isotherm	Parameters	303 K	313 K
Langmuir	Q_m_ (mg/g)	213.719	219.750
	K_L_ (L/mg)	0.1484	0.3160
	R^2^	0.9812	0.9910
	R_adj_^2^	0.9801	0.9905
Freundlich	K_F_ (mg^1−1/n^ L^1/n^ g^−1^)	44.5933	62.5156
	1/n	2.6316	2.8425
	R^2^	0.9499	0.9547
	R_adj_^2^	0.9469	0.9521
Dubinin–Radushkevich	Q_m_ (mg/g)	99.484	120.313
	K_DR_ (mol^2^/K^2^J^2^)	2 × 10^−7^	9 × 10^−8^
	E (KJ/mol)	2.236	3.333
	R^2^	0.7163	0.7951
	R_adj_^2^	0.6998	0.7849

**Table 3:** Comparison of adsorption performance of Alg@Mag/PPy NCs towards mercury(II) with other reported literature data.

Adsorbent	Isotherm Model	Kinetic Model	Percent Removal (%R)	Adsorption Capacity (mg/g)	pH	Conc. (mg/L)	Ref.
Magnetic PPy–Graphene oxide	Langmuir	P-so	99.0	400	7	100	[55]
Hydrazide-modified sodium alginate	Freundlich	P-fo	96.1	7.833 *	6	0.499 **	[81]
Polypyrrole-Fe_3_O_4_/Kaolin	Langmuir	P-so	-	317.1	7.2	50	[74]
Fe_3_O_4_@SiO_2_-S_4_	Langmuir	P-so	-	2.42 *	6	2 **	[75]
Urea-grafted alginate	Langmuir	P-so	-	200	5.5	0.25	[76]
Polypyrrole multilayer-laminated cellulose	Langmuir	P-so	96	31.689	6	100	[77]
Modified chitosan coated Fe_3_O_4_	Langmuir	P-so	97.3	96	5	25	[78]
Magnetic chitosan-thioglyceraldehyde	Langmuir	P-so	85	98	5	100	[79]
CoFe_2_O_4_@SiO_2_	Langmuir	P-so	-	149.3	7	20	[80]
Alg@Mag/PPy NCs	Langmuir	P-so	95.58	213.72	7	40	Our work

* mmol/g, ** mmol/L.

**Table 4:** Thermodynamic parameters for the adsorption of mercury(II) onto the Alg@Mag/PPy NCs.

T (K)	ΔS° (J/K/mol)	ΔH° (kJ/mol)	−ΔG° (kJ/mol)	R^2^
298	175.649	43.123	9.134	0.9923
303			10.125	
313			12.037	
323			13.489	

## Data Availability

The data presented in this study are available on request from the corresponding author.

## Supplementary Materials

The following supporting information can be downloaded at: https://www.mdpi.com/article/10.3390/polym15214285/s1, Figure S1: FTIR analysis of Alg and α-Fe_2_O_3_; Figure S2: XPS analysis (A) C1s (B) N1s (C) O1s (D) Fe2p (E) Hg4f of Alg@Mag/PPy NCs (1) Before (2) After adsorption; Figure S3: TEM images of α-Fe_2_O_3_ nanoparticles at 100 nm; Figure S4: BET analysis of Alg@Mag/PPy nanocomposite; Figure S5: Effect of initial mercury(II) ion concentration on the (A) adsorption capacity and (B) removal efficiency onto the Alg@Mag/PPy nanocomposite; Figure S6: Dubinin–Radushkevich isotherm models for the adsorption of mercury(II) onto the Alg@Mag/PPy nanocomposite.
