# Supplementary material for: Alginate Modified Magnetic Polypyrrole Nanocomposite for the Adsorptive Removal of Heavy Metal
Source: Polymers (Basel). 2023 Oct 31;15(21):4285. doi: 10.3390/polym15214285 (PMC10650565; doi:10.3390/polym15214285)
Supplement: Supplementary file 1 [file polymers-15-04285-s001.zip › polymers-2654386-supplementary.pdf]

Alginate Modified Magnetic Polypyrrole Nanocomposite for the Adsorptive Removal of Heavy Metal

Fouzia Mashkoor, Mohd Shoeb, Changyoon Jeong*

School of Mechanical Engineering, Yeungnam University, Gyeongsan, Gyeongbuk, 38541 Republic of Korea

Corresponding Author
Dr. Changyoon Jeong
Email: yoonni22@yu.ac.kr
School of Mechanical Engineering, Yeungnam University,
Gyeongsan 38541, Korea, Republic of Korea

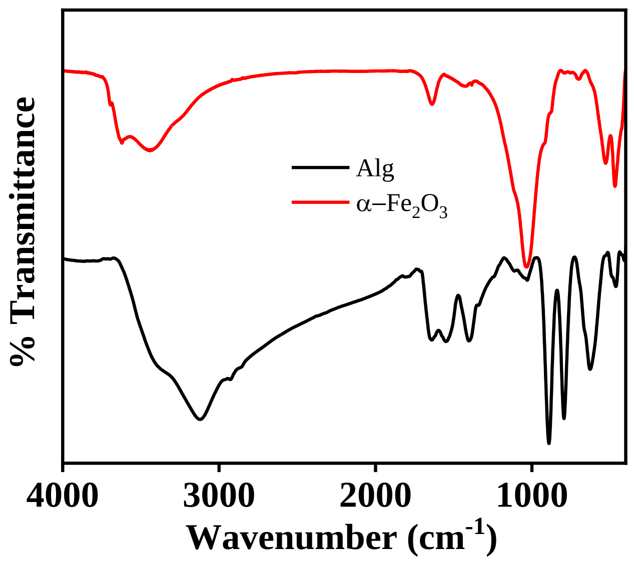

Figure S1. FTIR analysis of Alg and α-Fe_2O_3

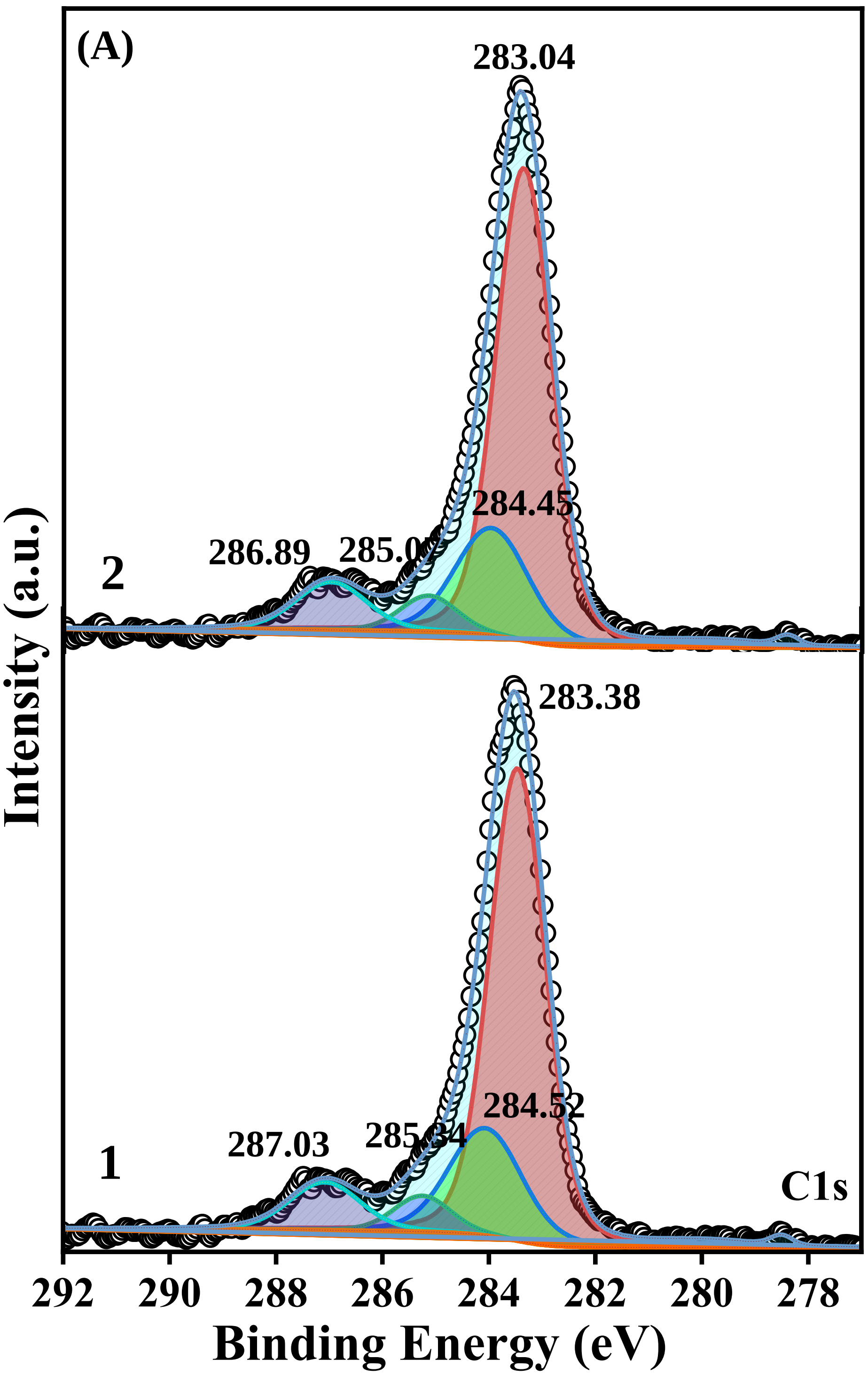

(A)
283.04
284.45
286.89
285.0
2
283.38
284.52
287.03
285.34
1
C1s
Intensity (a.u.)
292
290
288
286
284
282
280
278
Binding Energy (eV)

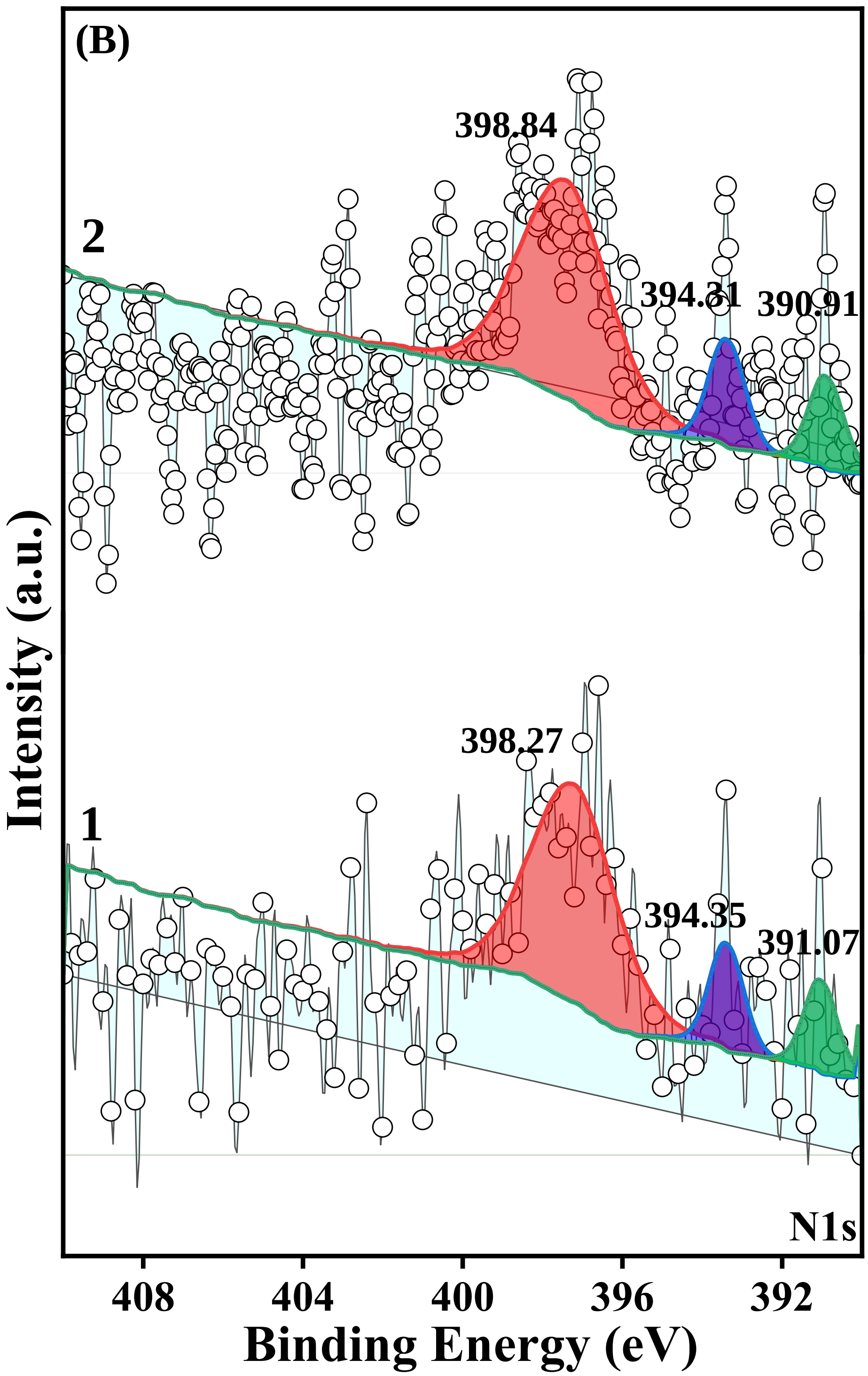

(B)
398.84
2
394.31
390.91
398.27
1
394.35
391.07
N1s
Intensity (a.u.)
408
404
400
396
392
Binding Energy (eV)

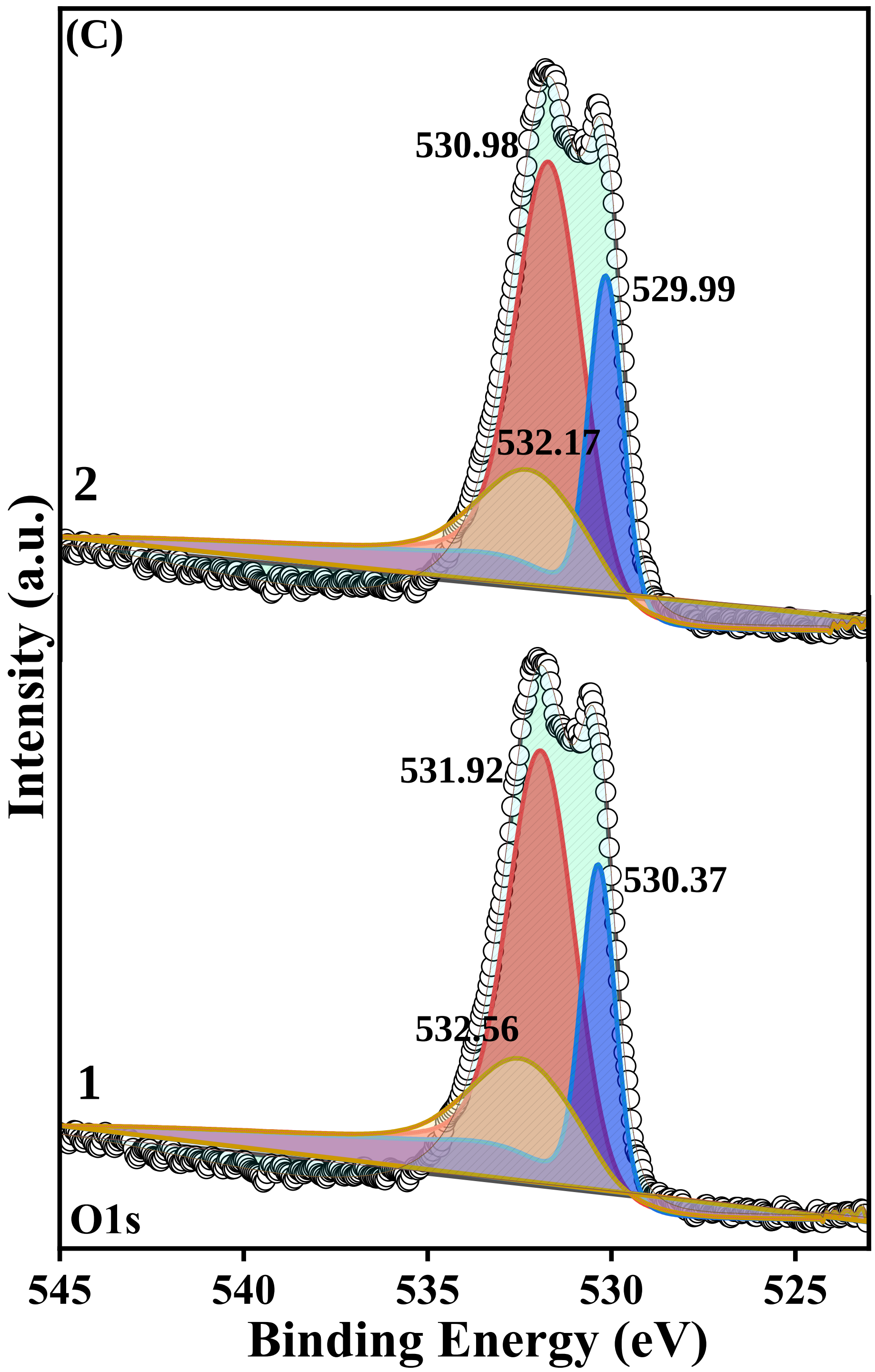

(C)
530.98
529.99
532.17
2
531.92
530.37
532.56
1
O1s
Intensity (a.u.)
545
540
535
530
525
Binding Energy (eV)

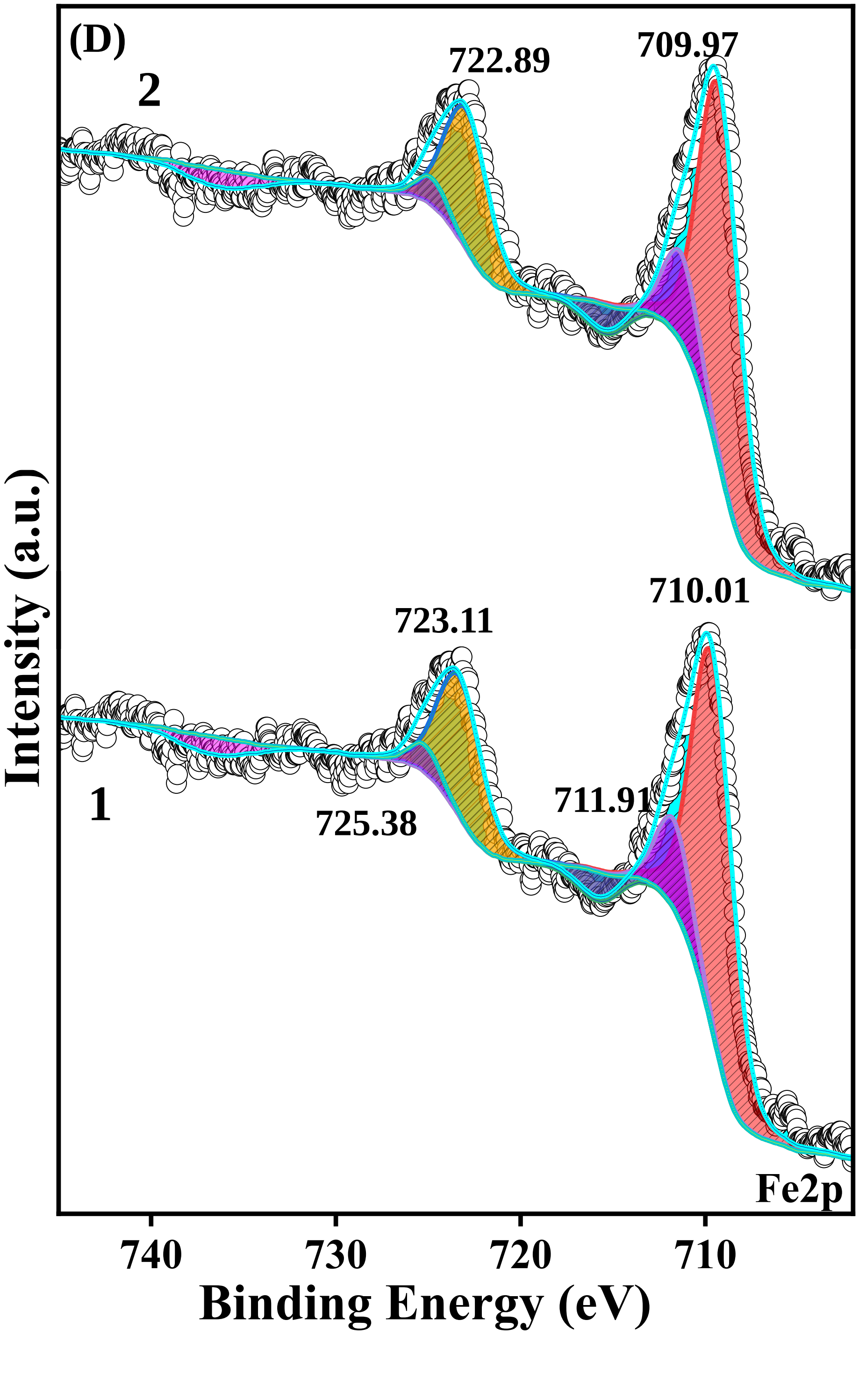
(D)
2
722.89
709.97
723.11
710.01
1
725.38
711.91
Fe2p
Intensity (a.u.)
740
730
720
710
Binding Energy (eV)

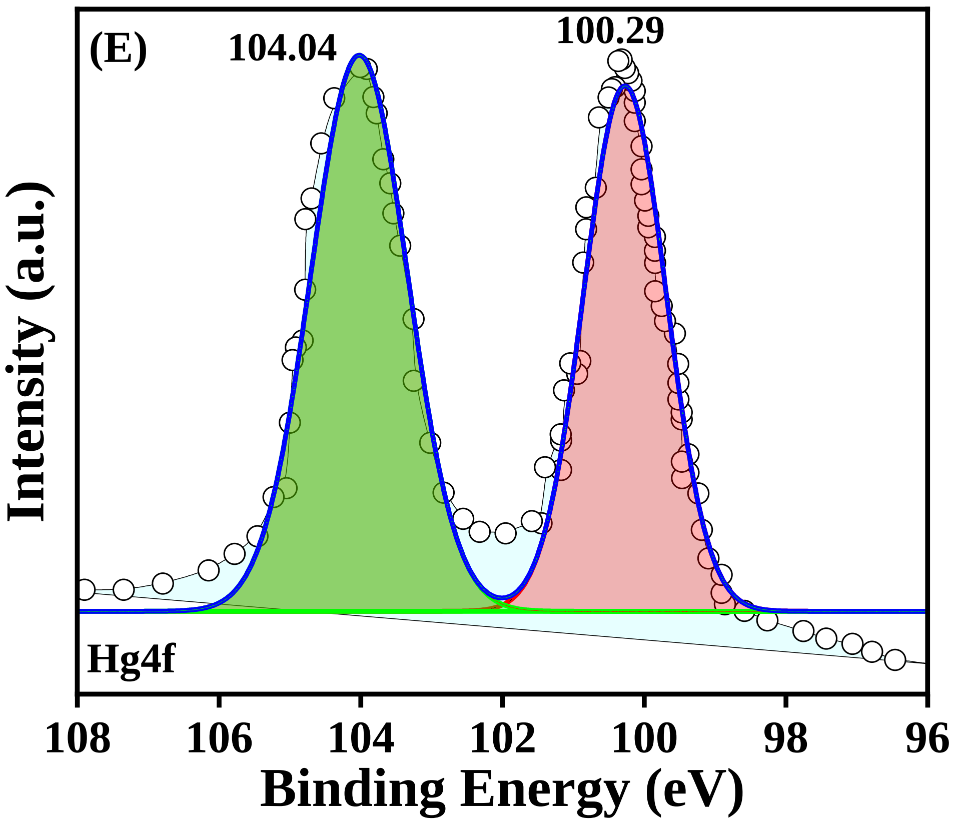

Figure S2. XPS analysis (A) C1s (B) N1s (C) O1s (D) Fe2p (E) Hg4f of Alg@Mag/PPy NCs (1) Before (2) After adsorption.

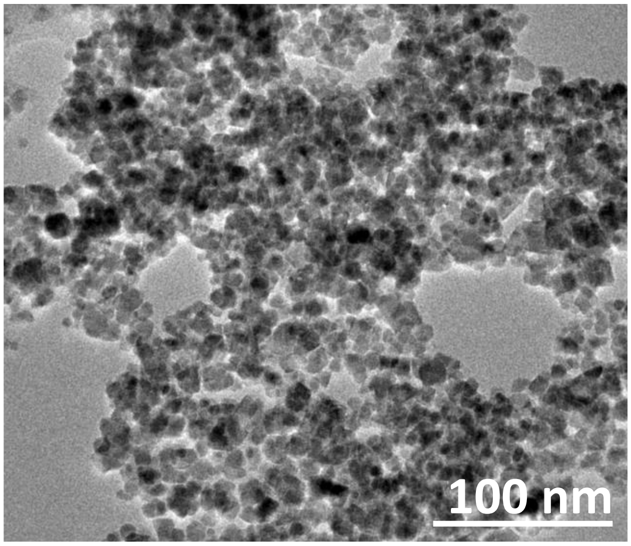

Figure S3. TEM images of α-Fe_2O_3 nanoparticles at 100 nm.

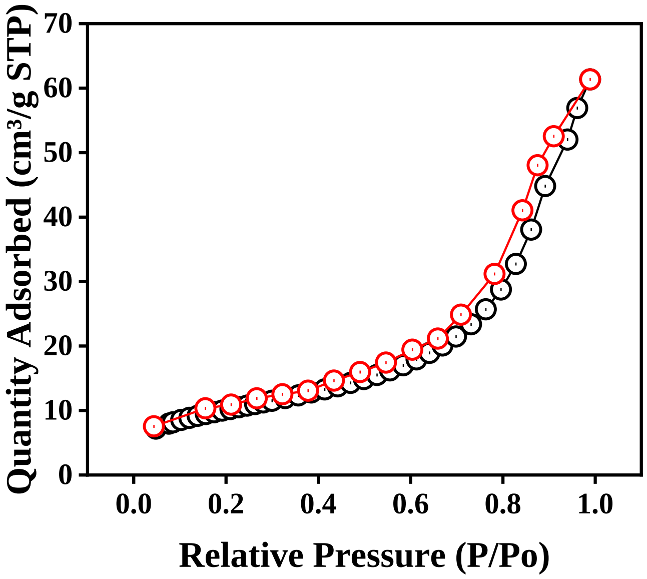

Figure S4. BET analysis of Alg@Mag/PPy nanocomposite.

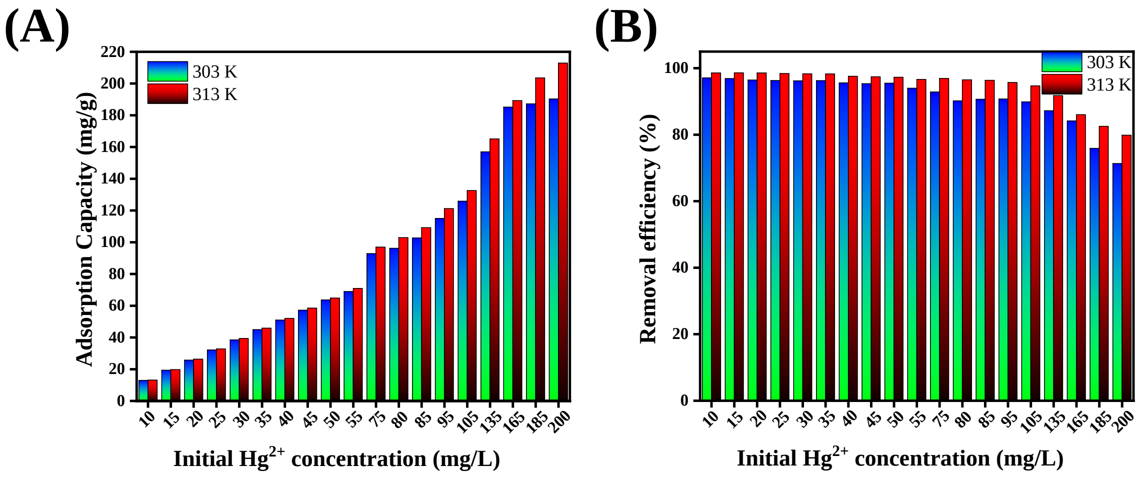

Figure S5. Effect of initial mercury(II) ion concentration on the (A) adsorption capacity and (B) removal efficiency onto the Alg@Mag/PPy nanocomposite.

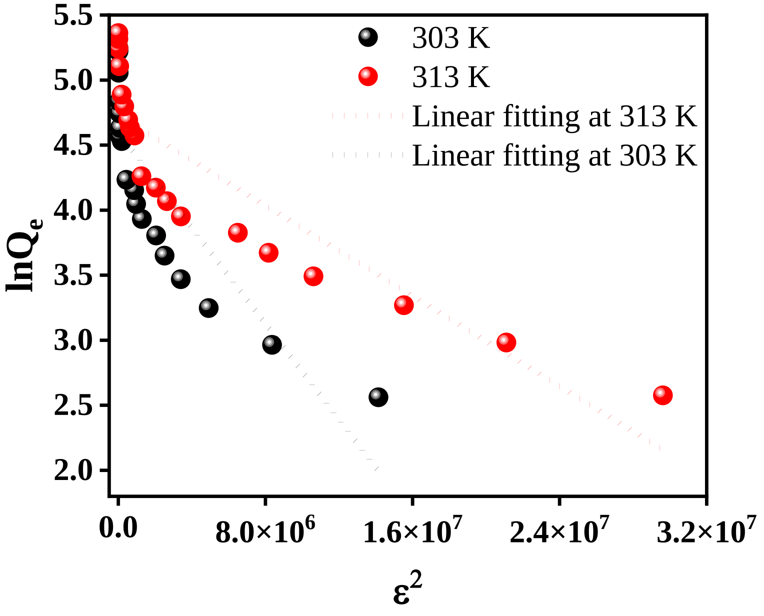

Figure S6. Dubinin–Radushkevich isotherm models for the adsorption of mercury(II) onto the Alg@Mag/PPy nanocomposite.

References

1. Xiong, C.; Wang, S.; Zhang, L. Selective recovery mechanism of Au(III) from an aqueous solution by trimethyl phosphate modified poly(glycidyl methacrylate). *Journal of the Taiwan Institute of Chemical Engineers* **2019**, *95*, 55-64, doi:https://doi.org/10.1016/j.jtice.2018.09.035.

2. Xiong, C.; Wang, S.; Hu, P.; Huang, L.; Xue, C.; Yang, Z.; Zhou, X.; Wang, Y.; Ji, H. Efficient selective removal of Pb (II) by using 6-aminothiouracil-modified Zr-based organic frameworks: from experiments to mechanisms. *ACS applied materials & interfaces* **2020**, *12*, 7162-7178.

3. Mashkoor, F.; Shoeb, M.; Mashkoor, R.; Anwer, A.H.; Zhu, S.; Jeong, H.; Baek, S.-S.; Jung, J.; Jeong, C. Synergistic effects of tungstate trioxide hemihydrate decorated reduced graphene oxide for the adsorption of heavy metals and dyes and postliminary application in supercapacitor device. *Journal of Cleaner Production* **2023**, *418*, 138067, doi:https://doi.org/10.1016/j.jclepro.2023.138067.
